# Associations of Fat Mass and Fat Distribution With Bone Mineral Density in Non-Obese Postmenopausal Chinese Women Over 60 Years Old

**DOI:** 10.3389/fendo.2022.829867

**Published:** 2022-01-25

**Authors:** Jingzheng Fan, Yuyan Jiang, Junlian Qiang, Bin Han, Qiang Zhang

**Affiliations:** ^1^ Department of Geriatrics, Tianjin Medical University General Hospital, Tianjin, China; ^2^ Department of Nuclear Medicine, Tianjin Medical University General Hospital, Tianjin, China; ^3^ Institute of Tianjin Geriatrics, Tianjin Medical University General Hospital, Tianjin, China

**Keywords:** bone mineral density, fat distribution, fat mass, lean mass, postmenopausal

## Abstract

**Background:**

Bone mineral density (BMD) loss is a major complication of menopause, and this loss is closely associated with Fat mass (FM). The relationship between FM, fat distribution (FD), and BMD in postmenopausal women, however, remains incompletely understood. The present study was thus developed to explore these associations between body fat accumulation, FD, and BMD among non-obese postmenopausal women over the age of 60.

**Methods:**

This was a cross-sectional analysis of 357 healthy postmenopausal women between the ages of 60.2 and 86.7 years. Dual-energy X-ray absorptiometry (DXA) was utilized to measure total and regional BMD as well as fat-related parameters including total FM, android and gynoid fat, body fat percentage (BF%), and total lean mass (LM) for all subjects. The android-to-gynoid fat ratio (AOI) was used to assess FD. Pearson’s correlation testing and multiple regression analyses were used to explore relationships among AOI, LM, FM, and BMD.

**Results:**

Both LM and FM were positively correlated with total and regional BMD in univariate analysis (all *P* < 0.01), whereas BMD was not significantly associated with AOI in any analyzed site other than the head. Multivariate linear regression models corrected for age, height, and years post-menopause, revealed a sustained independent positive relationship between FM and BMD (standard β range: 0.141 – 0.343, *P* < 0.01). The relationship between FM and BMD was unaffected by adjustment for LM (standard β range: 0.132 – 0.258, *P* < 0.01), whereas AOI had an adverse impact on BMD at most analyzed skeletal sites (total body, hip, femoral neck, arm, leg, and head) (standard β range: −0.093 to −0.232, *P* < 0.05). These findings were unaffected by using BF% in place of FM (standard β range: −0.100 to −0.232, *P* < 0.05).

**Conclusions:**

In this cohort of non-obese postmenopausal women over the age of 60 from China, total FM was positively associated with BMD, while AOI was negatively correlated with BMD. As such, a combination of proper weight gain and the control of central obesity may benefit the overall bone health of women after menopause.

## Introduction

Osteoporosis is a prevalent and often asymptomatic condition that commonly develops in women after menopause, contributing to significant reductions in quality of life over time. The diagnosis of osteoporosis is primarily made through measurements of bone mineral density (BMD) (1), which is in turn closely tied to body composition-related parameters including lean mass (LM) and fat mass (FM) (2). The relative degree to which LM and FM contribute to BMD, however, remains controversial. Some reports have found LM and FM to equally contribute to increases in bone mass among postmenopausal women (3–6). In contrast, other studies have suggested that FM has a significantly more pronounced beneficial impact on BMD relative to LM (7–10), while others have reported the exact opposite finding (11–13).

After menopause, woman commonly exhibit changes in body composition consisting of a reduction in gynoid fat together with an increase in central fat in the android region (14, 15). The android-to-gynoid fat ratio (AOI) has thus been reported to be a valuable indicator of central (visceral) fat accumulation that is correlated with BMD, but studies have yielded inconsistent findings regarding such a relationship (16–20). Shao et al., for example, found central fat accumulation to be negatively correlated with BMD (18), whereas Kapus et al. observed the opposite relationship (19). These contrasting results underscore the complex interplay between FM, fat distribution (FD), and BMD in postmenopausal women.

Most studies to date have either focused primarily on obese individual or have enrolled postmenopausal women without regard for their body weight, whereas few works have been selectively performed on healthy subjects with a body mass index (BMI) within non-obese limits (20–22). There have also been few studies to date exploring the relationships between FM, AOI, and BMD at different skeletal sites among elderly postmenopausal women (7). As such, this study was developed to examine the associations between FM, central FD, and total or regional BMD among non-obese postmenopausal Chinese women over the age of 60.

## Methods

### Subjects

In total, 357 non-obese (18.5 < BMI < 30 kg/m^2^) women 60 years of age or older were selected as a random sample from among patients at the Department of Geriatric Health Check-Up Centre, Tianjin Medical University General Hospital between January 2020 and August 2021. Participants were excluded from this study if they exhibited blood diseases, chronic renal diseases, pituitary disorders, thyroid diseases, a history of pathological fractures, known malignancies, rheumatoid arthritis, hypogonadism or were taking medicine with the potential to impact fat, lean mass, or bone metabolism (including calcium, vitamin D, hormone replacement therapy, oral contraceptives, anticonvulsants, diuretics, corticosteroid-containing asthma medications, oral anticoagulants, immunosuppressive drugs, or nonsteroidal ant-inflammatory drugs). None of the participants were heavy drinkers.

The Ethics Committee of the Tianjin Medical University General Hospital study approved the present study. With all subjects having provided written informed consent to participate.

### Data Collection

Data collected from patients through self-reported questionnaires and standardized interviews included: age (in years) and years since menopause (YSM). Participants were considered postmenopausal when they reported having experienced amenorrhea for 12 consecutive months.

Standard approaches were used to gather anthropometric data. Standing height (cm) was measured using a stadiometer accurate to within 0.1 cm accuracy, while body weight (kg) was measured using a portable electronic beam scale accurate to within 0.1 kg while participants were wearing only light clothing without shoes. Both parameters were measured twice. BMI was determined as follow: body weight (kg)/height^2^ (m^2^).

### Body Composition Analyses

**Figure 1:**
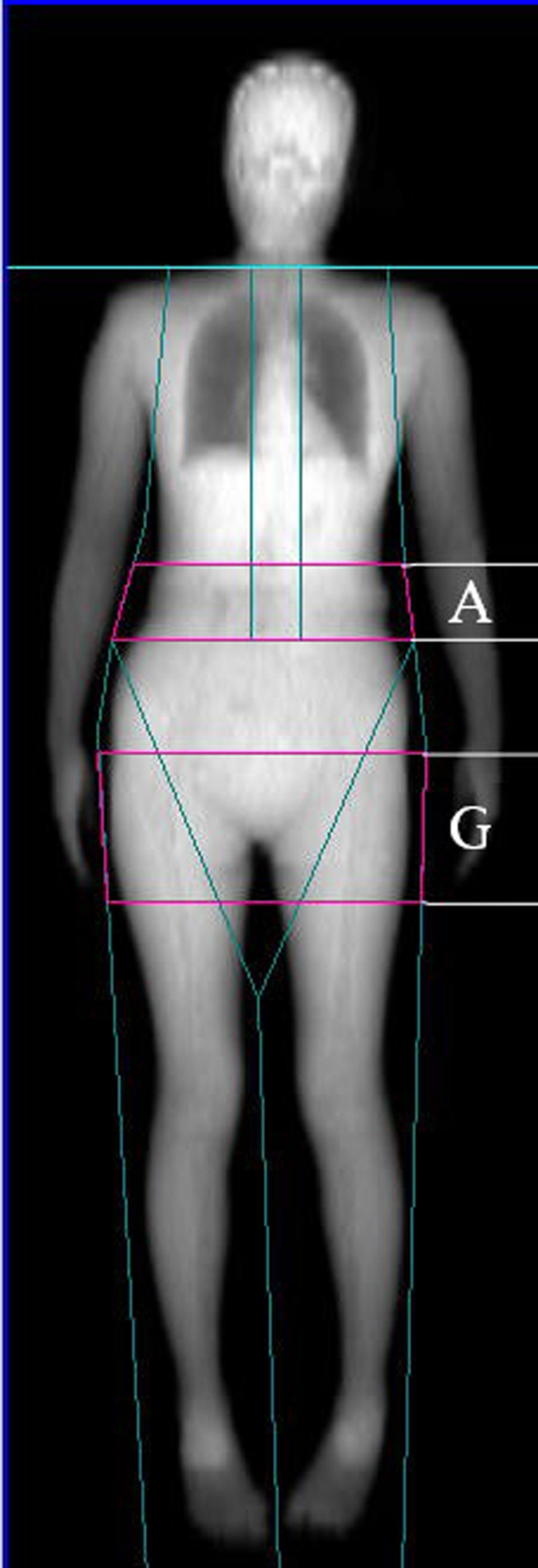
Dual-energy X-ray absorptiometry-based body composition measurement (A: android fat distribution ROI, G: gynoid fat distribution ROI).

FM, LM, body fat percentage (BF%), gynoid fat, android fat, and both total and regional BMD were assessed *via* whole-body DXA scanning (Software Version enCORE 13.40.038; Lunar Prodigy, GE Healthcare, USA). BF% was calculated as follows: BF%=total FM/total body weight x 100. Gynoid and android regions of interest (ROIs) were determined using the provided software base on the manufacturer’s instructions. The android ROI height was 20% of the distance from the pelvic horizontal cut line to the neck cut line, with the arm cut lines serving as lateral boundaries. The gynoid ROI height was two times that of the android ROI, with the leg cut lines serving as lateral boundaries, and the upper boundary being beneath the pelvic horizontal cut lines by 1.5 times the android ROI height (**Figure 1**). AOI was calculated by dividing the android FM by the gynoid FM. Regional BMD values were assessed for body regions including the head, ribs, legs, arms, lumbar spine, femoral neck, hip, and trunk. Prior to each measurement, densitometer standardization was performed. The absorptiometry machine was subject to daily quality control analysis. All DXA measurements were performed by a single trained technologist, yielding excellent precision for all measured parameters. The *in vivo* precision of such DXA-based body composition analyses has been demonstrated in prior reports, with coefficient of variation value of < 2% for all total and regional BMD measurements and < 3% for all body composition analyses. These measures were established using duplicate measures of the study cohort as in prior reports (18).

### Statistical Analysis

The Shapiro-Wilk test was used to assess the normality of data distributions. Continuous data are given as means ± standard deviation (SD). Pearson’s correlation analyses were used to explore relationships among different study variables, while multivariate linear regression models were utilized to evaluate relationships among BMD and AOI, LM, and FM, with age, height, and YSM serving as fixed covariates. In Model 1, the relationships between FM and AOI with total and regional BMD were assessed. Model 2 additionally explored the relationships between LM and total and regional BMD in a model incorporating FM and AOI. These regression analyses were then repeated, with BF% in place of FM. The results of these analyses are given as standardized regression coefficients. *P* < 0.05 was the threshold of significance, and data were analyzed using SPSS v16 (SPSS Inc., IL, USA).

## Results

### Descriptive Statistics

In total, 357 healthy, non-obese (BMI: 18.5 – 30) postmenopausal women were enrolled in the present study. Their demographic characteristics, anthropometric parameters, body composition-related findings, and BMD (total and regional) are compiled in **Table 1**. These participants had an average age of 69.1 years (range: 60.2 – 86.7 years). The mean number of years since menopause for these subjects was 18.6 (range: 6.1 – 38.7 years). The average FM of the overall study cohort was 20.36 kg, accounting for 34.7% of total body weight.

**Table 1:** Baseline characteristics of subjects.

Parameters	Mean ± SD	Range
Age (years)	69.12 ± 5.17	60.2-86.7
YSM (years)	18.61 ± 5.67	6.1-38.7
Height (cm)	157.57 ± 6.13	140-178
Body weight (kg)	58.26 ± 7.61	41-82
BMI (kg/m^2^)	23.45 ± 2.67	18.6-30
Body composition measures (kg)		
Total LM	35.45 ± 4.64	15.9-58.66
Total FM	20.36 ± 5.06	6.71-33.27
BF%	34.67 ± 5.76	11.77-51.58
Android fat	2.02 ± 0.59	0.22-3.89
Gynoid fat	3.28 ± 0.82	0.70-5.65
AOI	0.63 ± 0.16	0.22-1.34
Bone mineral density (g/cm^2^)		
Total body	1.006 ± 0.091	0.756-1.315
Total hip	0.833 ± 0.120	0.500-1.236
Femoral neck	0.773 ± 0.119	0.410-1.221
Lumbar spine	0.986 ± 0.171	0.426-1.586
Spine	0.928 ± 0.125	0.650-1.312
Arm	0.719 ± 0.090	0.537-1.107
Leg	1.043 ± 0.118	0.641-1.368
Trunk	0.803 ± 0.083	0.607-1.335
Rib	0.578 ± 0.052	0.453-0.764
Head	1.935 ± 0.281	1.315-3.401

YSM, years since menopause; BMI, body mass index; FM, fat mass;

BF%, body fat percentage; LM, lean mass; AOI, android to gynoid fat ratio.

### The Relationships Among Anthropometric, Body Composition, and BMD Parameters

In univariate analyses, higher LM, FM, and BF% were associated with increases in both total and regional BMD (total body, head, ribs, legs, arms, spine, lumbar spine, femoral neck, hips) (*r* = 0.199–0.499, all *P* < 0.01). In contrast, AOI was not significantly associated with total or regional BMD among postmenopausal women with the exception of head BMD (*r* = −0.140, *P* < 0.01). Both increased age and YSM were significantly negatively correlated with total and regional BMD values (all *P* < 0.05; **Table 2**). In contrast, height, body weight, and BMI were positively correlated with total and regional BMD values (all *P* < 0.05).

**Table 2:** Correlations between subject characteristics, body composition and total body. and regional BMD measurements.

	Total BMD	Hip BMD	FN BMD	LS BMD	Spine BMD	Arm BMD	Leg BMD	Trunk BMD	Rib BMD	Head BMD
Age (years)	-0.179^b^	-0.195^c^	-0.222^c^	-0.104^c^	-0.115^a^	-0.195^c^	-0.197^c^	-0.119^a^	-0.111^a^	-0.142^b^
YSM (years)	-0.183^b^	-0.207^c^	-0.227^c^	-0.116^c^	-0.106^a^	-0.206^c^	-0.199^c^	-0.135^a^	-0.114^a^	-0.124^b^
Height (cm)	0.329^b^	0.249^b^	0.346^c^	0.183^c^	0.211^c^	0.301^c^	0.322^c^	0.313^c^	0.260^c^	0.216^c^
BW (kg)	0.475^c^	0.373^b^	0.432^c^	0.368^c^	0.459^c^	0.418^c^	0.492^c^	0.558^c^	0.611^c^	0.282^b^
BMI (kg/m^2^)	0.305^b^	0.256^b^	0.252^c^	0.268^c^	0.360^c^	0.262^c^	0.328^c^	0.409^c^	0.497^c^	0.171^b^
LM (kg)	0.460^b^	0.320^b^	0.418^c^	0.369^c^	0.413^c^	0.421^c^	0.451^c^	0.499^c^	0.521^c^	0.282^c^
FM (kg)	0.315^b^	0.303^b^	0.300^c^	0.267^c^	0.357^c^	0.313^c^	0.410^c^	0.403^c^	0.499^c^	0.199^c^
BF%	0.157^b^	0.198^b^	0.156^b^	0.147^b^	0.215^c^	0.171^a^	0.275^c^	0.227^c^	0.328^c^	0.100^a^
AOI	-0.066	-0.031	-0.061	0.041	0.084	-0.036	0.010	0.023	0.102	-0.140^b^

^a^P < 0.05, ^b^P < 0.01, ^c^P < 0.001.

YSM, years since menopause; BW, body weight; BMI, body mass index; BMD, bone mineral density; FN, femoral neck; FM, fat mass; LS, Lumbar spine; BF%, body fat percentage; LM, lean mass; AOI, android to gynoid fat ratio.

Correlations between anthropometrics parameters and soft tissue-related variables are compiled in **Table 3**. These analyses revealed LM and FM to be positively correlated with the height, body weight, and BMI of postmenopausal women (all *P* < 0.001). While AOI was positively correlated with BMI and body weight, it was negatively correlated with height in this study cohort (all *P* < 0.05). BF% was positively associated with both body weight and BMI (all *P* < 0.001). Age or YSM were not correlated with FM, LM, AOI, or BF%.

**Table 3:** Correlation of soft tissue components with anthropometric parameters.

	Age (years)	YSM (years)	Height (cm)	Body weight (kg)	BMI (kg/m2)
Total LM (kg)	-0.005	-0.001	0.599^c^	0.719^c^	0.405^c^
Total FM (kg)	-0.065	-0.097	0.192^c^	0.787^c^	0.779^c^
BF%	-0.045	-0.084	-0.035	0.487^c^	0.601^c^
AOI	-0.025	0.001	-0.105^a^	0.174^b^	0.273^c^

^a^P < 0.05, ^b^P < 0.01, ^c^

P < 0.001.

YSM, years since menopause; BMI, body mass index; FM, fat mass; BF%, body fat percentage; LM, lean mass; AOI, android to gynoid fat ratio.

### Multivariate Analyses

Next, multivariate linear regression analyses were conducted to more fully explore the relationships among these different study variables (**Table 4**). Model 1 revealed a significant positive correlation between FM and both total and regional BMD values (total body, head, ribs, legs, arms, spine, lumbar spine, femoral neck, and hips) (standard β range: 0.141 to 0.343, all *P* < 0.001), while AOI was not significantly associated with any BMD parameters other than head BMD (r = -0.156, *P* < 0.01). Following adjustment for LM in Model 2, the positive correlations between FM and BMD remained significant (standard β range: 0.132 to 0.258, all *P* < 0.01). In addition, a significant relationship between LM and BMD was detected for all body regions (all *P* < 0.01), whereas AOI was significantly negatively correlated with head, leg, arm, femoral neck, hip, and total body BMD (standard β range: −0.093 to −0.232, all *P* < 0.05). When these analyses were repeated with BF% in place of FM, the results were largely the same (see **Table 5**), with BF% being significantly correlated with BMD. Following adjustment for LM, no change in the relationship between BF% and BMD was observed (standard β range: −0.100 to −0.232, all *P* < 0.05). A significant negative association between LM and BMD was also observed, while AOI was negatively correlated with head, femoral neck, arm, leg, hip, and total body BMD (standard β range: −0.100 to −0.232, *P* < 0.05).

**Table 4:** Regression analysis of FM, AOI, and LM with total body and regional BMD.

	Total BMD (g/cm^2^)			Hip BMD (g/cm^2^)			FN BMD (g/cm^2^)			LS BMD (g/cm^2^)			Spine BMD (g/cm^2^)		
	Sβ	*t*	*P*	Sβ	*t*	*P*	Sβ	*t*	*P*	Sβ	*t*	*P*	Sβ	*t*	*P*
Model 1: Without adjustment for LM															
FM (kg)	**0.244**	**5.074**	**<0.001**	**0.263**	**5.309**	**<0.001**	**0.227**	**4.759**	**<0.001**	**0.244**	**4.780**	**<0.001**	**0.322**	**6.503**	**<0.001**
AOI	-0.085	-1.750	0.081	-0.072	-1.440	0.151	-0.083	-1.711	0.088	0.012	0.238	0.812	0.049	0.981	0.327
Model 2: With adjustment for LM															
LM (kg)	**0.385**	**7.827**	**<0.001**	**0.210**	**4.038**	**<0.001**	**0.338**	**6.794**	**<0.001**	**0.275**	**5.238**	**<0.001**	**0.294**	**5.834**	**<0.001**
FM (kg)	**0.198**	**4.027**	**<0.001**	**0.245**	**4.721**	**<0.001**	**0.195**	**3.926**	**<0.001**	**0.182**	**3.469**	**0.001**	**0.248**	**4.904**	**<0.001**
AOI	**-0.184**	**-3.913**	**<0.001**	**-0.128**	**-2.566**	**0.005**	**-0.174**	**-3.664**	**<0.001**	-0.057	-1.119	0.264	-0.024	-0.485	0.628
	**Arm BMD (g/cm^2^)**			**Leg BMD (g/cm^2^)**			**Trunk BMD (g/cm^2^)**			**Rib BMD (g/cm^2^)**			**Head BMD (g/cm^2^)**		
	**Sβ**	***t***	***P***	**Sβ**	***t***	***P***	**Sβ**	***t***	***P***	**Sβ**	***t***	***P***	**Sβ**	***t***	***P***
Model 1: Without adjustment for LM															
FM (kg)	**0.141**	**4.569**	**<0.001**	**0.343**	**7.396**	**<0.001**	**0.261**	**5.539**	**<0.001**	**0.293**	**5.754**	**<0.001**	**0.206**	**3.946**	**<0.001**
AOI	0.013	0.267	0.790	0.008	0.169	0.866	0.001	0.010	0.992	-0.061	1.177	0.240	**-0.181**	**-3.506**	**0.001**
Model 2: With adjustment for LM															
LM (kg)	**0.452**	**9.293**	**<0.001**	**0.437**	**9.410**	**<0.001**	**0.192**	**2.997**	**0.003**	**0.195**	**3.690**	**<0.001**	**0.204**	**3.814**	**<0.001**
FM (kg)	**0.132**	**2.717**	**0.007**	**0.258**	**5.563**	**<0.001**	**0.214**	**5.539**	**<0.001**	**-0.246**	**4.663**	**<0.001**	**0.170**	**3.186**	**0.002**
AOI	**-0.093**	**-2.008**	**0.045**	**-0.093**	**-2.103**	**0.036**	0.001	0.010	0.992	-0.015	-0.284	0.777	**-0.232**	**-4.543**	**<0.001**

Models were adjusted for age, YSM, and height in postmenopausal women.

BMD, bone mineral density; FN, femoral neck; FM, fat mass; LS, Lumbar spine; LM, lean mass; AOI, android to gynoid fat ratio; Sβ, standardized coefficients β.

All significant values are shown in bold.

**Table 5:** Regression analysis of BF%, AOI, and LM with total body and regional BMD.

	Total BMD (g/cm^2^)			Hip BMD (g/cm^2^)			FN BMD (g/cm^2^)			LS BMD (g/cm^2^)			Spine BMD (g/cm^2^)		
	Sβ	*t*	*P*	Sβ	*t*	*P*	Sβ	*t*	*P*	Sβ	*t*	*P*	Sβ	*t*	*P*
Model 1: Without adjustment for LM															
BF%	**0.123**	**2.519**	**0.012**	**0.191**	**3.834**	**<0.001**	**0.138**	**2.868**	**0.004**	**0.153**	**2.971**	**0.003**	**0.200**	**3.931**	**<0.001**
AOI	-0.050	-1.016	0.310	-0.045	-0.889	0.374	-0.054	-1.109	0.268	0.040	0.767	0.443	0.086	1.667	0.096
Model 2: With adjustment for LM															
LM (kg)	**0.454**	**9.600**	**<0.001**	**0.298**	**5.968**	**<0.001**	**0.317**	**4.901**	**<0.001**	**0.339**	**6.843**	**<0.001**	**0.381**	**7.946**	**<0.001**
BF%	**0.121**	**2.578**	**0.010**	**0.191**	**3.851**	**<0.001**	**0.143**	**3.014**	**0.003**	**0.131**	**2.648**	**0.008**	**0.173**	**3.595**	**<0.001**
AOI	**-0.182**	**-3.779**	**<0.001**	**-0.132**	**-2.604**	**0.010**	**-0.143**	**-2.821**	**0.005**	-0.059	-1.142	0.254	-0.025	-0.494	0.621
	**Arm BMD (g/cm^2^)**			**Leg BMD (g/cm^2^)**			**Trunk BMD (g/cm^2^)**			**Rib BMD (g/cm^2^)**			**Head BMD (g/cm^2^)**		
	**Sβ**	***t***	***P***	**Sβ**	***t***	***P***	**Sβ**	***t***	***P***	**Sβ**	***t***	***P***	**Sβ**	***t***	***P***
Model 1: Without adjustment for LM															
BF%	**0.139**	**2.807**	**0.005**	**0.268**	**5.670**	**<0.001**	**0.104**	**2.074**	**0.039**	**0.169**	**3.263**	**0.001**	**0.118**	**2.278**	**0.023**
AOI	0.039	0.778	0.437	0.008	0.169	0.866	0.040	0.790	0.430	-0.095	1.819	0.070	**-0.156**	**-3.016**	**0.003**
Model 2: With adjustment for LM															
LM (kg)	**0.500**	**10.843**	**<0.001**	**0.531**	**12.095**	**<0.001**	**0.229**	**3.634**	**<0.001**	**0.282**	**5.590**	**<0.001**	**0.264**	**5.159**	**<0.001**
BF%	**0.127**	**2.768**	**0.006**	**0.249**	**5.703**	**<0.001**	**0.196**	**3.979**	**<0.001**	**0.154**	**3.048**	**0.002**	**0.113**	**2.216**	**0.027**
AOI	**-0.100**	**-2.136**	**0.033**	**-0.106**	**-2.387**	**0.018**	-0.038	-0.714	0.476	-0.018	0.337	0.736	**-0.232**	**-4.462**	**<0.001**

Models were adjusted for age, YSM, and height in postmenopausal women.

BMD, bone mineral density; FN, femoral neck; FM, fat mass; LS, Lumbar spine; BF%, body fat percentage; LM, lean mass; AOI, android to gynoid fat ratio; Sβ, standardized coefficients β.

All significant values are shown in bold.

## Discussion

These analyzes revealed total FM to be positively correlated with BMD for all analyzed skeletal regions, whereas AOI, serving as a readout for central FD, was negatively correlated with BMD for most skeletal regions following adjustment for age, height, YSM, total FM, and total LM among non-obese postmenopausal Chinese women over 60 years of age.

Consistent with our expectations, we found that most analyzed anthropometric parameters such as age and YSM were strongly correlated with BMD, both of which were negatively correlated with total and regional BMD values. In contrast, these BMD indices were positively correlated with the height, body weight, and BMI of study participants, although these relationships became less clear upon in-depth analyses of the relationships between anthropometric variables and soft tissue parameters. While some soft tissue parameters were positively correlated with height, weight, and BMI, others were negatively correlated with these variables or not clearly related to them. This suggests that the interplay between FD, anthropometric factors, and body fat accumulation has the potential to be beneficial or harmful with respect to BMD. It is thus vital that these anthropometric parameters be controlled for when evaluating relationships between bone mass and fat. However, prior studies have indicated that using body weight or BMI to correct for the effects of LM or FM on BMD has the potential to result in incorrect conclusions given that both LM and FM are tightly correlated with overall body weight (with correlation coefficients of 0.79 and 0.72 for FM vs. body weight and LM vs. body weight, respectively) (23). The incorporation of both total FM and body weight into a regression model has the potential to lead to inaccurate conclusions as a consequence of mathematical coupling (24–26). In contrast, height has been shown to be a more appropriate readout to use when seeking to control for body size (3). LM also has the potential to be leveraged as a variable for adjustment when assessing relationships between fat and bone mass (26). In the present analysis, we thus utilized age, height, and YSM as fixed covariates and total LM as an additional covariate for adjustment in our multivariate linear regression analyses exploring the associations between FM and BMD.

We observe a positive relationship between BMD and total FM among postmenopausal women, in line with prior reports (3, 7, 9). This relationship may be attributable to the elevated gravitational force associated with increased weight, in turn contributing to improvements in BMD (26). However, given that total FM accounts for a relatively small fraction of overall body weight, such gravitational forces are unlikely to fully explain the interplay between FM and BMD. Other research suggests that adipocytes can produce hormones including adiponectin, leptin, insulin, and adipocytic estrogens, all of which can impact bone metabolism *via* the endocrine pathway, thus potentially contributing to these results (27–30). These hormones may play a protective role, stimulating osteoblastogenesis and inhibiting the resorption of established bone tissue mediated by osteoclasts (31).

While increases in bone mass were observed with rising total FM in this analysis, BMD values for most analyzed regions were negatively correlated with central fat accumulation, as measured based on AOI, in non-obese postmenopausal elderly women. These findings are consistent with those from other studies suggesting that DXA-based AOI values are negatively correlated with bone health (16–18). This result may be attributable to a few underlying mechanisms. For one, adipose tissue sources can release high levels of inflammatory cytokines such as TNF-α or IL-6, thus contributing to bone loss and decreased BMD (32–34). Secondly, free fatty acid secretion from the visceral adipose tissue can inhibit insulin receptor expression, thereby contributing to the incidence of insulin resistance (35). Third, the osteoblastic and adipocytic differentiation of mesenchymal stem cells (MSCs) have been shown to be negatively correlated (36). The same mechanisms that are active in the bone marrow may thus be ties to the interplay between bone and central fat deposits.

In this study, we additionally observed strong positive correlations between LM and BMD in all analyzed body sites, with these correlations generally being stronger than those observed for FM. This suggests that muscle-mediated mechanical loads have a more robust beneficial impact on BMD as compared to FM in postmenopausal women (3, 9, 11, 12).

There are multiple strengths to the present study. For one, our research subjects were recruited from among a single well-defined population of individuals over 60 years of age of a specific ethnicity. Second, this study is among the few to have explored the association between central FD and BMD among non-obese postmenopausal women. Third, we assessed both total BMD and the regional BMD at multiple sites including the head, spine, lumbar spine, arms, legs, trunk, ribs, hips, and femoral neck, and we utilized DXA-based AOI as a measure for central FD rather than using alternative metrics such as the waist-to-thigh or waist-to-hip ratio.

There are a number of limitations to the present study. For one, this study was cross-sectional in design, thus precluding our ability to draw causal inferences pertaining to the relationships between FM, AOI, LM, and BMD. Secondly, no premenopausal women were included in this study, and all study participants were Chinese, thus limiting the degree to which these data are generalizable. Third, while we adjusted for age, height, and YSM when assessing the relationships between FM, FD, and BMD, we did not take other potential confounding variables such as serum sex hormone levels, vitamin D levels, dietary composition, smoking, or socioeconomic status into consideration when conducting multivariable regression analyses.

In conclusion, the results of this analysis suggest that FD and FM are associated with BMD among postmenopausal Chinese woman over the age of 60, even after adjusting for age, height, YSM, and LM. AOI can serve as an indicator of central FD, and was found to be negatively associated with both total and regional BMD, whereas total FM exhibit a positive relationship with BMD at all analyzed body sites, suggesting that it may serve as a protective factor. Total LM exhibited results consistent with total FM, thus suggesting that proper weight gain with appropriate control of central obesity may be beneficial to bone health among postmenopausal women. These data emphasize the important of regular physical activity, which can reduce central obesity even in the absence of weight loss while also reducing age-related muscle atrophy and increasing mechanical loading of the skeletal system (37).

## Data Availability Statement

The original contributions presented in the study are included in the article/supplementary material. Further inquiries can be directed to the corresponding author.

## Ethics Statement

The studies involving human participants were reviewed and approved by the Ethics Committee of the Tianjin Medical University General Hospital. The patients/participants provided their written informed consent to participate in this study. Written informed consent was obtained from the individual(s) for the publication of any potentially identifiable images or data included in this article.

## Author Contributions

JF and YJ contributed equally to this work and share first authorship. JF and YJ designed the investigation. JQ and BH conducted the investigation and collected data. YJ performed the statistics. QZ wrote the main manuscript. All authors contributed to the article and approved the submitted version.

## Funding

This work was funded by the National Natural Science Foundation of China (Grant No. 92163213, 81970085 and 82000844), and Science and Technology Talent Cultivation Project of Tianjin Municipal Health Commission (Grant No. KJ20216), and the Tianjin science and sechnology plan project (Grant No. 17ZXMFSY00080).

## Conflict of Interest

The authors declare that the research was conducted in the absence of any commercial or financial relationships that could be construed as a potential conflict of interest.

## Publisher’s Note

All claims expressed in this article are solely those of the authors and do not necessarily represent those of their affiliated organizations, or those of the publisher, the editors and the reviewers. Any product that may be evaluated in this article, or claim that may be made by its manufacturer, is not guaranteed or endorsed by the publisher.
